# Thermal plasticity in postembryonic life history traits of a widely distributed Collembola: Effects of macroclimate and microhabitat on genotypic differences

**DOI:** 10.1002/ece3.3333

**Published:** 2017-09-05

**Authors:** Sagnik Sengupta, Torbjørn Ergon, Hans Petter Leinaas

**Affiliations:** ^1^ Department of Biosciences University of Oslo Oslo Norway; ^2^ Centre for Ecological and Evolutionary Synthesis Department of Biosciences University of Oslo Oslo Norway

**Keywords:** differential selection pressure, latitudinal cline, microevolution, phenotypic plasticity, seasonal time constraints, springtails, thermal adaptation, trait mean

## Abstract

Life history traits in many ectotherms show complex patterns of variation among conspecific populations sampled along wide latitudinal or climatic gradients. However, few studies have assessed whether these patterns can be explained better by thermal reaction norms of multiple life history traits, covering major aspects of the life cycle. In this study, we compared five populations of a Holarctic, numerically dominant soil microarthropod species, *Folsomia quadrioculata*, sampled from a wide latitudinal gradient (56–81°N), for growth, development, fecundity, and survival across four temperatures (10, 15, 20, and 25°C) in common garden experiments. We evaluated the extent to which macroclimate could explain differences in thermal adaptation and life history strategies among populations. The common garden experiments revealed large genotypic differences among populations in all the traits, which were little explained by latitude and macroclimate. In addition, the life history strategies (traits combined) hardly revealed any systematic difference related to latitude and macroclimate. The overall performance of the northernmost population from the most stochastic microclimate and the southernmost population, which remains active throughout the year, was least sensitive to the temperature treatments. In contrast, performance of the population from the most predictable microclimate peaked within a narrow temperature range (around 15°C). Our findings revealed limited support for macroclimate‐based predictions, and indicated that local soil habitat conditions related to predictability and seasonality might have considerable influence on the evolution of life history strategies of *F. quadrioculata*. This study highlights the need to combine knowledge on microhabitat characteristics, and demography, with findings from common garden experiments, for identifying the key drivers of life history evolution across large spatial scales, and wide climate gradients. We believe that similar approaches may substantially improve the understanding of adaptation in many terrestrial ectotherms with low dispersal ability.

## 1 INTRODUCTION

In ectotherms, ambient temperature is a major determinant of variations in life history traits, and therefore of individual performance (Angilletta, Steury, & Sears, 2004; Roff, 2002). Consequently, organisms from contrasting climates are likely to show variation in thermal adaptation, and comparative studies of conspecific populations represent an important approach for elucidating the underlying processes (Barton, Sunnucks, Norgate, Murray, & Kearney, 2014; Gotthard & Nylin, 1995; Sinclair, Williams, & Terblanche, 2012; Trotta et al., 2006). Both mean climate conditions and climate variability affect thermal adaptation (Condon, Cooper, Yeaman, & Angilletta, 2014; Nilsson‐Örtman, Stoks, De Block, & Johansson, 2012). However, not all traits may show clinal patterns of variation owing to the evolutionary constraints imposed by the combined effect of direct selection, genetic correlations, and physiological interlinks (see Angilletta, Wilson, Navas, & James, 2003; van Heerwaarden & Sgrò, 2011).

Phenotypic plasticity is an important means of coping with climate variability (Ellers & Stuefer, 2010; Pigliucci, 2005; Pigliucci, Murren, & Schlichting, 2006), and it might differ among conspecific populations. Therefore, it is important to investigate the differences among population in both trait mean and slope (phenotypic plasticity) of thermal reaction norms in order to understand the drivers of adaptive variation in life history traits (Janion, Leinaas, Terblanche, & Chown, 2010). Thus, comparison of reaction norms for selected life history traits among populations sampled along a macroclimate or latitudinal gradient is a common approach for understanding thermal adaptation (Klepsatel et al., 2013; Liefting, Hoffmann, & Ellers, 2009; Trotta et al., 2006). Correlation of trait means and plasticity with macroclimate variables is often emphasized (e.g., Barton et al., 2014; Liefting et al., 2009; Trotta et al., 2006).

It is generally assumed that organisms adapted to more variable temperature conditions would show plastic responses to perform well across a wide range of temperatures (Amarasekare & Johnson, 2017; Klepsatel et al., 2013; Oomen & Hutchings, 2016). Consequently, the large temperature variation often occurring in the habitats of terrestrial ectotherms at higher latitudes may favor more generalist type of thermal responses than conspecifics from lower latitudes (see Danks, 1999, 2004; Klepsatel et al., 2013; Liefting et al., 2009). Similarly, it is often argued that populations from higher latitudes would have larger trait means and phenotypic plasticity for optimally utilizing the shorter growing seasons (Conover & Schultz, 1995).

Several detailed comparisons of thermal reaction norms, focussing on few selected life history traits, have demonstrated that conspecific populations evolve in response to mean temperature and temperature variability (Berger, Walters, & Blanckenhorn, 2014; Condon et al., 2014; Klepsatel et al., 2013). Studies based on this approach have emphasized the important role of macroclimate variables in thermal adaptation (James, Azevedo, & Partridge, 1995; Liefting et al., 2009; Trotta et al., 2006) and suggested that habitat characteristics related to microclimate might complicate clinal patterns to varying extents (van Heerwaarden, Lee, Overgaard, & Sgrò, 2014; Klepsatel et al., 2013; Nilsson‐Örtman et al., 2012; Scriber, Elliot, Maher, McGuire, & Niblack, 2014). Consequently, evaluation of the extent to which macroclimate may explain population‐level variation is considered a prerequisite for identifying the key drivers of thermal adaptation (van Heerwaarden et al., 2014; Manenti, Sørensen, & Loeschcke, 2017). Further, different traits may vary in their responses to selection (e.g., Liefting et al., 2009). Therefore, a comparison of multiple traits across ecologically relevant temperatures can potentially improve our understanding of the important underlying processes. However, such comparisons are laborious and complex, and therefore restricted to few model organisms with short generation times (e.g., Schmidt, Matzkin, Ippolito, & Eanes, 2005; Trotta et al., 2006). Consequently, the importance of including multiple life history traits to better explain the evolution of clinal patterns across wide geographical and climatic ranges has remained relatively understudied.

Collembolans are important members of the soil fauna, with great effects on litter decomposition and nutrient turnover rates in most terrestrial systems (Lavelle & Spain, 2001; Rusek, 1998). They are easy to maintain in culture, and convenient model organisms for life history studies (Birkemoe & Leinaas, 1999; Liefting & Ellers, 2008; Stam, van de Leemkule, & Ernsting, 1996). Relatively few studies, however, compare life history traits of conspecific Collembolan populations from a wide range of climates (but see Stam, 1997; Sengupta, Ergon, & Leinaas, 2016). The Collembolan species *Folsomia quadrioculata* (Tullberg, 1871) is widely distributed in temperate and arctic regions and numerically dominant in many habitat types (Chimitova, Chernova, & Potapov, 2010; Deharveng & Lek, 1995; Fjellberg, 1994), and well‐suited for addressing questions related to the evolution of life history strategies across a wide range of climates. Earlier, a comparison of embryonic life history traits of several arctic and temperate populations of this species had shown marked genotypic variation, but only small effects of latitude and macroclimate (Sengupta et al., 2016). However, in contrast to the eggs, the animals (i.e., postembryonic stages) are mobile, and therefore, likely to differ considerably from the embryonic stages in their responses to temperature. To address this possibility, we compared thermal effects on growth, development, reproduction, and survival in five chosen populations of *F. quadrioculata* in common garden experiments. Together, these traits cover the major aspects of the vital rates of a population, and include important trade‐offs, such as age and size at maturity.

We compared five populations from Denmark (56°N) in the south to the high arctic Little Slate Island (81°N) of Svalbard in the north (Table 1) to evaluate the effect of large differences in macroclimate on temperature responses of life history traits. In order to evaluate possible effects of microclimate, we chose the two high arctic and three temperate populations, based on available information on seasonal variation and predictability of ground temperature. The two high arctic sites, Little Slate Island (LSI) and Ellef Ringnes Island (ERI), are both very cold but differ greatly in stochasticity, notably in occasional periods of warm spells (McAlpine, 1965; Savile, 1961; Sengupta et al., 2016). The two temperate sites near Oslo, a dense coniferous forest (OF) and an open field (Ås) differ in patterns of diurnal and seasonal temperature variation owing to difference in vegetation cover (Bjor, 1972; Schnug, Jensen, Scott‐Fordsmand, & Leinaas, 2014). The third temperate population DK, from Denmark, originated from an area with mild winters, allowing continuous reproduction throughout most of the year (Rasmussen, Nielsen, & Hansen, 1982), compared to a 1‐year life cycle with distinct phenology in the Oslo area (Leinaas, 1978).

**Table 1:** Location, year of collection, and climate information of the sites of origin of the five populations of *Folsomia quadrioculata*. Macroclimate (air temperature based) variables used to compare postembryonic life history traits are listed under “Predictor variables” (in bold). *T*_S_ = mean air temperature from June to August, dd = annual heat sum above 0°C (day degree). Microclimate (soil habitat based) information is summarized under “Soil microclimate.” “Soil exposure” indicates the likelihood of soil temperature being influenced by insolation and wind. “Growing season type” summarizes the soil temperature conditions and length of the active season based on duration of snow cover, frost, and summer temperature. “Predictability” indicates how the soil temperature conditions vary during the growth season and between years. For sources of air temperature data, see footnotes. For further details about “Soil microclimate,” see Appendix S1 and Sengupta et al. (2016). Soil temperature records were not available

Population	Area	Year of sampling	Predictor variables			Vegetation	Soil microclimate		
			Co‐ordinates	*T*_S_ (°C)	dd (day degrees)		Soil exposure	Growing season type	Predictability
Little Slate Isl. (LSI)^a^	NE, Svalbard	2007	**80.821; 20.360**	**2.2**	**203.4**	Arctic tundra	Small island, highly exposed	Short cold	Highly unpredictable favorable warm spells
Ellef Ringnes Isl. (ERI)^b^	NW, Canada	1999	**78.794; −103.551**	**1.2**	**200**	Arctic tundra	Exposed	Short cold	Constantly cold
Oslo Forest (OF)^c^	Norway	2007	**59.988; 10.476**	**16.0**	**2751.2**	Forest (spruce)	Near 100% ground vegetation cover	Moderately long, fairly warm	Predictable, fairly warm summer, cool and humid in spring and autumn
Ås^d^	Norway	2010	**59.659; 10.754**	**15.3**	**2536.8**	Grass field	No canopy cover	Moderately long, fairly warm	Higher diurnal temperature fluctuations in summer than OF
Denmark (DK)^e^	Denmark	2007	**56.288; 10.469**	**15.2**	**2814.0**	Forest (beech)	Fairly exposed in early spring before leafing	Activity all year, fairly warm summers	Temperature changes with time of the year

^a^Norwegian Meteorological Institute—data of nearby Phipps Island (see Birkemoe & Leinaas, 2001).

^b^Records of Isachsen weather station in www.isachsen.climatemps.com.

^c^Norwegian Meteorological Institute—data of Oslo (Blindren).

^d^Norwegian Meteorological Institute—data of Ås.

^e^Data of Aarhus in www.mitrejsevejr.dk.

To evaluate the hypothesis that large‐scale climatic variation is a major driver of thermal adaptation in postembryonic life history traits of *F. quadrioculata*, we estimated the extent to which the observed variation in thermal reaction norms of these traits across populations is explained by differences in macroclimate among the sites of origin. Further, as an alternative explanation, we evaluated how habitat characteristics, which are known to affect the microclimate, might explain deviations from predictions based on macroclimate.

## 2 MATERIAL AND METHODS

### 2.1 Study organism, sites, and climate

*Folsomia quadrioculata* is a soil litter‐dwelling (hemi‐edaphic) Collembolan. It is widespread in the Holarctic, inhabiting diverse habitats (Deharveng & Lek, 1995; Hågvar, 2010; Hertzberg, Leinaas, & Ims, 1994; Ponge, 1993, 2000; Sengupta et al., 2016). It shows indeterminate growth, reproduces sexually, and life cycles range from several generations per year in continental Europe (Grégoire‐Wibo, 1976; Haarløv, 1960) to more than a 1‐year life cycle in the arctic (Birkemoe & Sømme, 1998; Hertzberg, Yoccoz, Ims, & Leinaas, 2000).

We compared five populations sampled from 56–81°N (relevant site characteristics in Table 1). Owing to the considerably large difference in season length among sites, we included both mean temperature of three summer months (*T*_S_), and annual heat sum above 0°C (dd) as proxies for temperature condition of the peak growing season and annual physiological time available for growth and reproduction, respectively. Detailed information on soil microclimate is given in Appendix S1.

### 2.2 Experimental design

The stock cultures of each population were founded with 100–200 Collembolans, extracted without heating from 5 to 10 randomly collected soil samples. Both stock rearing and experiments used plastic culture boxes (diameter = 3.4 cm, height = 3 cm), with plastic lids and moist bottom of plaster of Paris mixed with charcoal (for detailed information on pre‐experimental treatment of the stock cultures see Appendix S2 and Sengupta et al., 2016). For temperature treatments (10, 15, 20, and 25°C), we used climate incubators (Sanyo MIR 553, Osaka, Japan). Temperature inside the cabinets was recorded on an hourly basis (Hobo Data Logger U12, Onset, MA, USA), and inspected once a week. The thermostat was re‐adjusted if the mean temperature of a cabinet diverged by more than ±0.2°C from the set temperature. High humidity was maintained in all the cultures (see Sengupta et al., 2016). All the stock cultures were kept at 15°C. The Collembolans were fed ad libitum with pieces of bark, covered by a dark crust dominated by cyanobacteria, growing on three adjacent trees at the university campus. Food was renewed once a week at 10 and 15°C, and twice a week at 20 and 25°C (to prevent fungal growth). This study, being a part of a more extensive comparison of arctic Collembolans, used constant light (55 cm Osram 14 W, intensity: 8 μmol m^−2^ s^−1^) as the standardized light regime. This was also used for the temperate populations, as all environmental variables, except temperature, had to be standardized (Sengupta et al., 2016).

All the experiments were performed from 2011 to 2013, i.e., 3–5 years (approximately 9–15 generations in stock cultures) after collection. The only exception to this was the ERI population, which had been collected in 1999 during an expedition to the remote Ellef Ringnes Island in arctic Canada (see Table 1 for collection dates). Owing to the extreme climate of ERI, we believed that its inclusion in this study would contribute to our understanding of the evolution of life history traits of *F. quadrioculata*. A couple of years after being collected, this population was shown to differ markedly in embryonic development and reproductive biology from conspecific populations sampled from Svalbard (Sengupta, 2015; Sengupta et al., 2016). In addition, we tested the effect of the duration of pre‐experimental culturing on all the focal populations, but found no significant effect (see Appendix S2).

**Figure 1:**
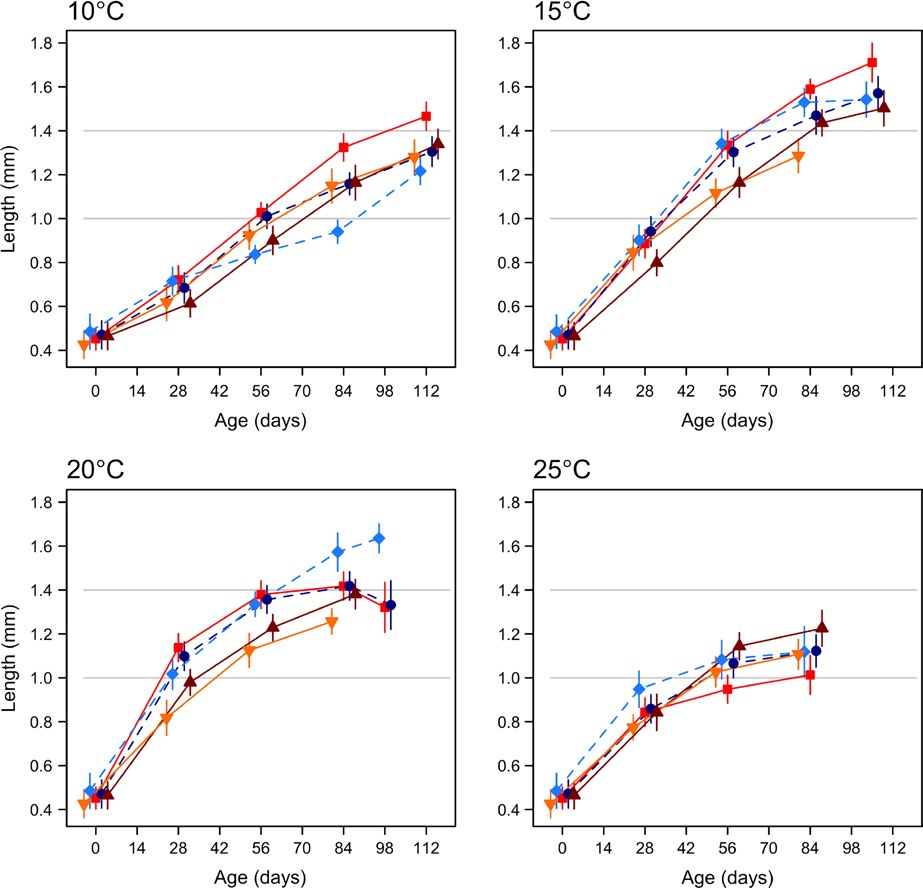
Growth trajectories of five populations of *Folsomia quadrioculata* at 10, 15, 20, and 25°C (followed up to 112, 105, 98, and 84 days respectively). Populations are indicated by different colors and shapes: dark blue circles–Little Slate Island (LSI), light blue diamonds–Ellef Ringnes Island (ERI), bright red squares–Oslo Forest (OF), dark red triangles–Ås, and yellow inverted triangles–Denmark (DK). Point estimates and 95% CI are fitted predictions from linear mixed effects models with population × temperature × age interactions and replicate boxes as random effects. The gray lines correspond to 1.0 and 1.4 mm. Note that DK was measured unto 84 days at 15 and 20°C; Ås was measured up to 84 days at 20°C

The experimental animals originated from eggs laid and developed at 15°C. Eggs were picked randomly from the stock cultures, placed in clean egg boxes, and inspected daily. This species thrives only when reared in groups, and each experimental culture (replicate box) was therefore started with even aged groups of 15–20 animals. The replicate boxes were randomly selected for the different temperature treatments. Growth patterns were studied at all the four temperatures, starting with 12–15 replicate boxes per treatment (population and temperature), depending on temperature. Growth was recorded at 28‐day intervals, by randomly terminating three replicates from each treatment for body length measurements. The experiments were maintained for 84–112 days depending on temperature (Figure 1). These experimental procedures were based on previous studies of this species (Sengupta, 2015; Sengupta et al., 2016).

### 2.3 Trait measurements

All individuals from the selected replicate boxes were killed in 70% ethanol and stretched by heating to 70°C. Body lengths were measured from photographs using Leica Application Suite (LAS v.3.7, Leica Microsystems). Analyzes of growth were based on body length measurements of individuals. Asymptotic size was estimated from mean length of the individuals in each replicate box. The number of eggs produced and animal mortality was recorded weekly. As a proxy for age at first reproduction, we used the mean age of each replicate between the two consecutive weekly recordings, during which the first eggs were laid. Fecundity was estimated from the cumulative sum of eggs produced per living animal in each culture box, as sex determination of the thousands of animals used in these experiments was not feasible. Mortality was recorded by counting and removing all dead animals throughout the experiments.

### 2.4 Analysis

#### 2.4.1 Comparison of thermal reaction norms

Growth trajectories were analyzed by plotting mean body length (estimated from linear mixed effects models accounting for random variation among replicate boxes) against age. Population‐specific asymptotic sizes were estimated by fitting mean length of individuals (*l*_*i*_) in each replicate box *i* (of population *p*(*i*)) at the age of termination (*t*_*i*_) to the growth model of von Bertalanffy (1938): $E\left(l_{i}\right)=L_{\infty ,p(i)}\left(1-e^{\left(-K_{p(i)}*\left(t_{i}-t_{0,p(i)}\right)\right)}\right)$, where the parameters are respectively population‐specific asymptotic size (*L*_∞, *p*(*i*)_), a population‐specific growth rate (*K*_*p*(*i*)_), and hypothetical time at length zero *t*_0, *p*(*i*)_. The model was fitted using “nlsList” function (package “nlme,” Pinheiro, Bates, DebRoy, Sarkar & R Core Team, 2014) in R version 3.2.2 (R Development Core Team, 2015), which assumed a normally distributed residual error.

At 10°C, where growth was slow, we were not able to estimate asymptotic size in two populations even after extending the duration of the experiment to 140 days. Hence, for this treatment, we also compared body length measurements at 112 days using log‐linear mixed effects model (Table S1).

Population‐specific estimates of the age at first reproduction were obtained from a log‐linear model with population, temperature, and their interaction as fixed effects, fitted to the proxy for age at first reproduction (see Section 2.3). Population‐specific estimates of size (body length) at first reproduction were obtained as predictions from linear mixed effects models (separate models for each treatment, i.e., population and temperature) with age as fixed effect and replicate box as random effects, fitted to body length measurements just before and after the estimated age at first reproduction.

Juvenile growth rates were estimated as percent increase in length per day (parallel to estimation of relative growth rate), from log‐linear mixed effects models. Separate models for each treatment, with age as fixed effect and replicate box as random effects, were fitted between hatchling size and length measurements from the replicate boxes terminated closest to first reproduction. An extension of this log‐linear function (Table S2) estimated plasticity of this trait over the linear part of the reaction norms (10–20°C, see Section 3).

Population‐level cumulative egg production at the age of 84 days was compared using log‐linear models fit by generalized least squares (GLS) (“gls” function, REML fit, package “nlme”). The model assumed separate variances per treatment using the “varIdent” function. This was selected by comparing Akaike Information Criterion (AIC) values of several models following the protocol of Zuur, Ieno, Walker, Saveliev, and Smith (2009). Population, temperature, and their interaction were included as fixed effects. This analysis, however, excluded the ERI population, which did not reproduce at 10°C (see Section 3).

Cox's proportional hazards models were used to estimate differences between populations in mortality (hazard) rates at each temperature (10–25°C). Due to very high survival in all the populations at 10 and 15°C (>92%), the rest of the analysis was restricted to the two highest temperatures (20 and 25°C). Models with population, temperature, and population × temperature interactions as fixed effects estimated differences in slope across these two temperatures. These analyzes were performed using package “survival” (Therneau, 2015).

#### 2.4.2 Effect of population covariates on thermal reaction norms

We focused on estimating percentage‐wise change in elevation and slope of reaction norms per unit change in population covariates (latitude, *T*_S_, and dd), and percentage of total observed variance explained. However, owing to the way the different traits had to be recorded (see Section 2.3), this could only be estimated for age at first reproduction, fecundity, and survival (20 and 25°C). To estimate the effects of the population covariates on age at first reproduction and fecundity, we used log‐linear mixed effects models, which included a fixed effect of treatment temperature, and a random population effect. The strongly correlated covariates were included, one at a time, in separate models in interaction with the fixed effect. Following the approach of Nakagawa and Schielzeth (2013), variance explained by the fixed effects (marginal *R* ^2^), the entire model (conditional *R* ^2^), and the random population effect was computed. Residuals were assumed to be normally distributed. The 95% confidence intervals were calculated by parametric bootstrap with 10,000 independent simulations (function confint.merMod and bootMer). For survival, the covariates were included in three separate Cox proportional hazards models in interaction with the effect of treatment temperature (only 20–25°C). Because the estimates of asymptotic size, size at first reproduction, and juvenile growth rate were not replicate specific, this analysis was limited to Pearson's product moment correlations at each temperature, and interpretation of the 95% confidence intervals of the point estimates.

All the statistical analyzes were carried out using R 3.2.2 (R Development Core Team, 2015). All (log‐)linear mixed effects models were fitted using restricted maximum likelihood (REML) in package “lme4” (Bates, Maechler, Bolker, & Walker, 2015). Assumptions of all (log‐)linear models were validated by graphical investigation of residual plots. No major violations of normality and homogeneity were detected.

## 3 RESULTS

### 3.1 Growth

In all populations, the growth curves were steepest in the initial phase (28–56 days, depending on temperature), and increasingly so from 10 to 20°C (Figure 1). The subsequent, gradual leveling out started at younger ages as temperature increased (Figure 1). There were, however, great differences among populations, and their relative performance varied with temperature and age (Figure 1). The Oslo Forest (OF) population reached the largest size of the five populations at 10–15°C, and grew larger than the other two temperate populations (Ås and DK) throughout most of the experiment at 20°C. At 25°C, however, OF had least growth of all the populations and was particularly smaller than the other population from the Oslo area (Ås). The northernmost Little Slate Island (LSI) population showed a similar growth curve as that of the southernmost DK at 10°C but was more similar to OF at 15 and 20°C. The other high arctic population (ERI) showed an irregular growth curve at 10°C and reached by far the largest size of all populations at 20°C.

**Figure 2:**
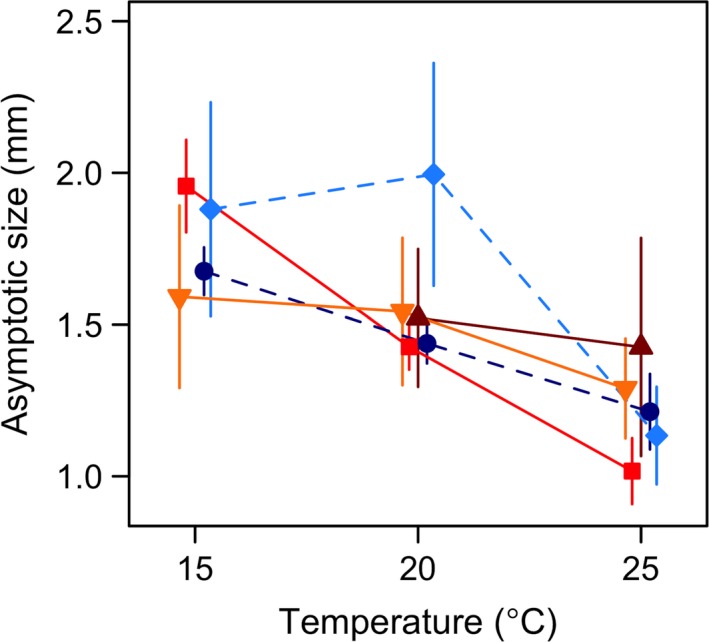
Population‐specific reaction norms for asymptotic size plotted against temperature. The estimates are based on von Bertalanffy's growth model fitted to mean length of the animals in each replicate box. Whiskers indicate 95% CI. Populations are indicated by different colors and shapes: dark blue circles–Little Slate Island (LSI), light blue diamonds–Ellef Ringnes Island (ERI), bright red squares–Oslo Forest (OF), dark red triangles–Ås, and yellow inverted triangles–Denmark (DK). Due to the shape of the growth curve of the Ås population at 15°C, the asymptotic size was overestimated and the 95% CI was very wide (and hence not plotted here; values shown in Table S3). Estimates for 10°C are shown Table S3

The asymptotic size showed a general tendency to decrease with increasing temperature (Figure 2; Table S3), but there were some significant differences among populations (nonoverlapping 95% CI in Figure 2). Estimation of asymptotic size at 10°C was complicated by weak tendencies of the growth curves to level out with age until the experiments ended (Figure 1). Accordingly, it could be estimated only for three populations (LSI, OF, and DK) at this temperature, and the 95% CI for these estimates were very wide, with extensive overlap (Table S3), although by the age of 112 days at 10°C, all the other populations were significantly smaller than OF (Table S1; Figure 1). At 15°C, the growth curve of the Ås population was not well described by von Bertalanffy's model, resulting in very wide 95% CI at this temperature (Table S3). This population was, therefore, not included in Figure 2 (at 15°C). Nonetheless, even at this temperature, Ås was significantly smaller than OF at the age of 105 days (nonoverlapping 95% CI in Figure 1). The Oslo Forest population (OF) showed the steepest decline in asymptotic size across temperatures among the five populations, whereas the northernmost high arctic LSI and the southernmost temperate DK populations were least affected by temperature (Figure 2). Correlation (Pearson's coefficient) of population‐specific asymptotic size with latitude, *T*_S_, and dd was not significant (*p* > .37, see Table S4).

**Figure 3:**
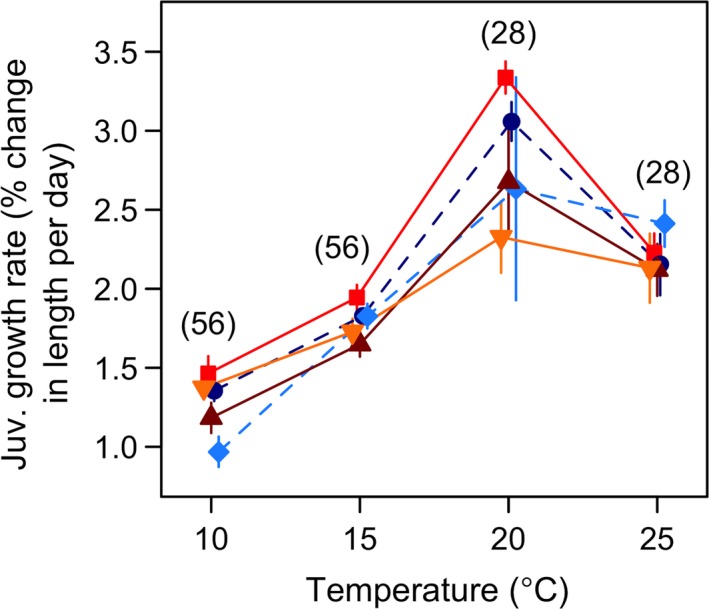
Population‐specific reaction norms for juvenile growth rate. Juvenile growth rate was estimated as the percentage increase in body length per day from the day of hatching to the age (in days; indicated within parentheses) closest to age at first reproduction. Populations are indicated by different colors and shapes: dark blue circles–Little Slate Island (LSI), light blue diamonds–Ellef Ringnes Island (ERI), bright red squares–Oslo Forest (OF), dark red triangles–Ås, and yellow inverted triangles–Denmark (DK). Point estimates and 95% CI are fitted predictions from log‐linear mixed effects models (see Section 2.4)

In all the populations, juvenile growth rate increased with temperature up to 20°C, and then decreased toward 25°C (Figure 3). Some populations differed distinctly from each other at 10 and 15°C (nonoverlapping 95% CI, Figure 3), but the largest differences among populations occurred at 20°C. OF had among the fastest juvenile growth rates from 10 to 20°C, which was much faster than that of the other population from the Oslo area (Ås) (nonoverlapping 95% CI in Figure 3). The Danish population (DK) resembled Ås, although it was less affected by temperature (Figure 3). DK differed significantly from OF and ERI in slope (Figure 3; Table S2). In contrast to the other three populations, the high arctic ERI and the Danish populations were little affected by increase in temperature from 20 to 25°C (see 95% CI in Figure 3). Population differences in juvenile growth rate were not significantly correlated with latitude, *T*_S_, and dd (Figure 3; Table S2).

### 3.2 Age and size at first reproduction

**Figure 4:**
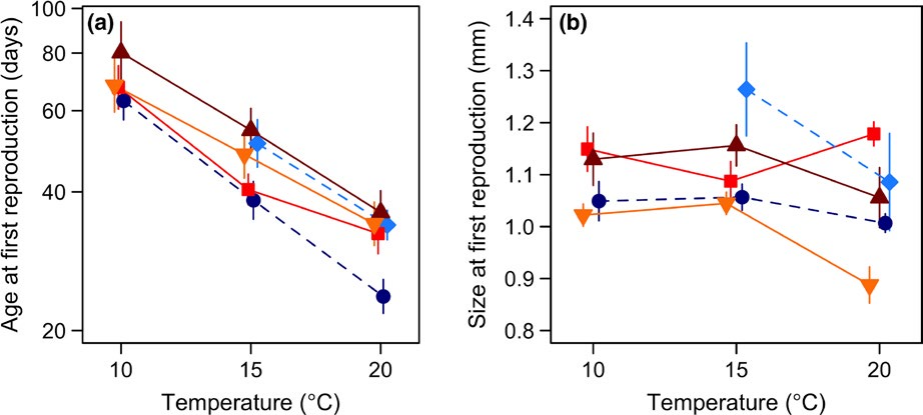
(a) Age at first reproduction (estimated from log‐linear models) plotted against temperature. A logarithmic scale has been used on the vertical axis, (b) Size at first reproduction plotted against temperature. Size at first reproduction was predicted from linear mixed effects models fitted to the growth trajectories (see Section 2.4). Populations are indicated by different colors and shapes: dark blue circles–Little Slate Island (LSI), light blue diamonds–ERI, bright red squares–Oslo Forest (OF), dark red triangles–Ås, and yellow inverted triangles–Denmark (DK). Whiskers indicate 95% CI. The Ellef Ringnes Island (ERI) population did not reproduce at 10°C and none of the populations reproduced at 25°C

Age at first reproduction decreased strongly with increasing temperature (Figure 4a). This trend was seen in all populations, but a slightly significant population × temperature interaction effect indicated some differences in the slopes; i.e., phenotypic plasticity (Figure 4a, log‐linear model: population × temperature: *F*_4, 89_ = 2.5, *p* = .05). LSI had the steepest slope, whereas the gentlest slope was seen in DK. OF had the most irregular curve. In addition, there was great variation in the population‐specific estimates across temperatures (i.e., trait means), ranging from the high arctic LSI starting reproduction at the youngest age to the late reproducing open field population (Ås). The latter started reproduction nearly 20 days later than LSI throughout the temperature range; i.e., at 28%–52% older age (Figure 4a). Great variations in age at first reproduction were present among the two arctic and among the three temperate populations (Figure 4a). Consequently, effects of latitude, *T*_S_, and dd on age at first reproduction were small, with only interactions of the covariates with treatment temperature (i.e., effect on plasticity) being significant (Table 2). The fixed effects (temperature, covariate, and interaction effects together) explained 72%–73% of the total variance. A considerable fraction of the variance in this trait was explained by the random population effect (13%, Table 2). Very little change in $R_{f}^{2}$ or conditional *R* ^2^ upon addition of any of the covariates, suggested that the covariates explained very little of total variance among populations (Table 2).

**Table 2:** Summary of the relative effects of population covariates on age at first reproduction estimated from four log‐linear mixed effects models. Latitude, temperature of growing season (*T*_s_), and heat sum (dd) were included as covariates in separate models in interaction with treatment “Temperature” (i.e., “Plasticity”). All 95% CI given in parenthesis are based on percentiles from 10,000 parametric bootstrap simulations. $R_{f}^{2}$ = percentage of variance explained by fixed effects (marginal *R* ^2^), $R_{r}^{2}$ = percentage of variance explained by random “Population” effects, conditional *R* ^2^ = total percentage of variance explained by the model. Effect sizes with 95% CI that exclude zero are highlighted in bold. The 95% range refers to relative (%) difference between 97.5 and 2.5 percentile of the random effects and residual distributions. Note that none of the populations reproduced at 25°C

Fixed effects	Effect size			Variance components			
				Among populations		Residuals	Conditional *R* ^2^
	% increase	Plasticity^a^	$R_{f}^{2}$	95% range	$R_{r}^{2}$	95% range	
Temperature (per °C)	−**7.86 (**−**8.51,** −**7.14)**		72.7 (61.0, 85.0)	68.8 (15.8, 145.6)	13.0 (2.0, 28.3)	73.3 (60.1, 86.5)	85.7 (82.6, 90.5)
**Covariates (in separate models)**							
Latitude (per 10°N)	11.67 (−5.92, 35.00)	−**0.98 (**−**1.66,** −**3.13)**	72.9 (55.0, 86.1)	80.7 (11.2, 188.0)	15.0 (0.5, 36.3)	69.7 (57.2, 82.8)	87.9 (83.7, 92.0)
*T*_S_ (per °C)	−1.91 (−4.58, 0.87)	**0.15 (0.04, 0.26)**	71.5 (52.9, 85.8)	86.5 (12.6, 203.2)	16.5 (0.8, 38.4)	70.0 (57.5, 83.7)	88.0 (83.6, 92.0)
dd (per 100 day degree)	−1.04 (−2.39, 0.34)	**0.09 (0.02, 0.14)**	71.9 (53.1, 85.8)	85.2 (30.0, 186.2)	16.2 (0.8, 38.8)	69.8 (57.0, 83.3)	88.0 (83.7, 92.0)

^a^Effect of interaction between treatment temperature and the population covariates (introduced one at a time). Values shown are percentage increase in age at first reproduction per unit increase in population covariate and temperature (i.e., change in slope of the reaction norms).

Size at first reproduction was the only trait that did not show systematic effects of temperature (Figure 4b). Interestingly, the northernmost LSI and southernmost DK populations had the smallest estimates across temperatures. Moreover, at 20°C, DK was much smaller than all the other populations. In comparison, the high arctic ERI had the largest estimated size at first reproduction at 15°C (Figure 4b). Sizes at first reproduction did not show significant correlation with latitude, *T*_S_, and dd at any of the temperatures (*p* > .47, see Table S4).

### 3.3 Fecundity

**Figure 5:**
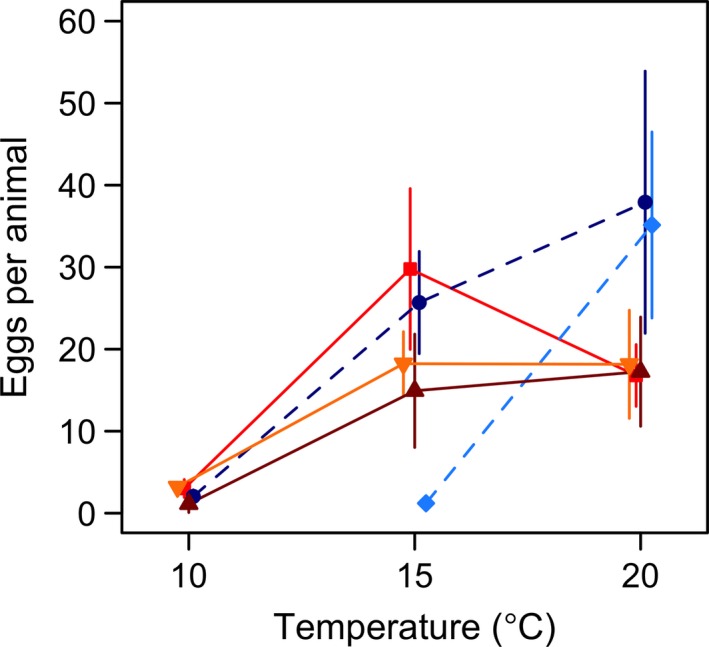
Fecundity (cumulative sum of eggs per animal alive) at the age of 84 days plotted against temperature. Populations are indicated by different colors and shapes: dark blue circles–Little Slate Island (LSI), light blue diamonds–Ellef Ringnes Island (ERI), bright red squares–Oslo Forest (OF), dark red triangles–Ås, and yellow inverted triangles–Denmark (DK). The ERI population did not reproduce at 10°C and none of the populations reproduced at 25°C. Point estimates and 95% CI are fitted predictions from generalized least squares (GLS) models (see Section 2.4)

Fecundity was strongly affected by temperature. The populations showed little reproduction at 10°C, varied considerably at both 15 and 20°C, and did not reproduce at all at 25°C (Figures 5 and S1). Counter intuitively, the two high arctic populations (LSI and ERI) had high fecundity at 20°C by the age of 84 days (Figure 5). ERI, from the site with the coldest growing season, differed strongly from the other populations in producing very few eggs at 15°C and none at 10°C (Figures 5 and S1). The two populations of the Oslo area (OF and Ås) differed from each other in population × temperature interactions at the age of 84 days (GLS model: difference = 43.2% (95% CI: 21.6%–68.6%), *t* = 4.3, *p* < .001). OF showed a peak in egg production at 15°C, whereas Ås was little affected by a temperature change from 15 to 20°C, similar to DK (Figure 5). The effects of latitude, *T*_S_, and dd on fecundity until the age of 84 days were not significant (Table 3). The fixed effects (temperature, covariate, and interaction effects together) explained 34% of the total variance (Table 3).

**Table 3:** Summary of the relative effects of population covariates on cumulative egg production until the age of 84 days, estimated from log‐linear mixed effects models. Latitude, temperature of growing season (*T*_s_), and heat sum (dd) were included as covariates in separate models in interaction with treatment “Temperature” (i.e., “Plasticity”). All 95% CI given in parenthesis are based on percentiles from 10,000 parametric bootstrap simulations. $R_{f}^{2}$ = percentage of variance explained by fixed effects (marginal *R* ^2^), $R_{r}^{2}$ = percentage of variance explained by random. “Population” effects, conditional *R* ^2^ = total percentage of variance explained by the model. Effect sizes with 95% CI that exclude zero are highlighted in bold. The 95% range refers to relative (%) difference between 97.5 and 2.5 percentile of the random effects and residual distributions

Fixed effects	Effect size			Variance components			
				Among populations		Residuals	Conditional *R* ^2^
	% Increase	Plasticity^a^	$R_{f}^{2}$	95% range	$R_{r}^{2}$	95% range	
Temperature (per °C)	**36.9 (23.5, 50.9)**		35.9 (27.1, 47.1)	1058 (0, 13595)	8.9 (0, 29.6)	43779 (13872, 135098)	44.8 (33.5, 60.7)
**Covariates (in separate models)**							
Latitude (per 10°N)	−8.7 (−82.0, 375.0)	2.0 (−7.5, 12.5)	34.0 (22.9, 46.4)	2037.9 (0, 20563)	13.2 (0, 42.2)	45869 (15053, 176934)	47.2 (33.2, 66.7)
*T*_S_ (per °C)	0.2 (−24.2, 32.5)	−0.2 (−1.9, 1.4)	33.9 (22.9, 46.8)	2089 (0, 70733)	13.3 (0; 41.9)	46046 (14080, 144690)	47.2 (33.2, 66.5)
dd (per 100 day degree)	1.5 (−12.2, 17.7)	−0.2 (−1.1, 0.7)	33.8 (22.8, 46.6)	2200 (0, 67413)	13.7 (0, 43.1	45699 (13758, 141955)	47.5 (33.4, 66.5)

^a^Effect of interaction between treatment temperature and the population covariates (introduced one by one). Values shown are percentage increase in egg production per unit increase in population covariate and temperature.

### 3.4 Mortality

**Figure 6:**
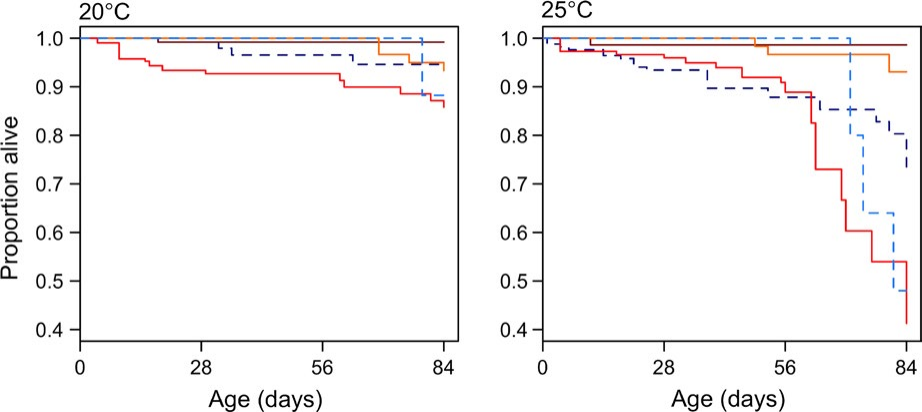
Survival plots based on Cox proportional hazards model for five populations at 20 and 25°C until the age of 84 days. Populations are indicated by different colors: dark blue–Little Slate Island (LSI), light blue–Ellef Ringnes Island (ERI), bright red–Oslo Forest (OF), dark red–Ås, and yellow– Denmark (DK). Due to high survival (>90%) at 10 and 15°C, survival at these two temperatures has not been plotted here

There was little mortality in any of the populations at 10 and 15°C throughout the duration of this study (<8%), with no significant difference among populations. However, the differences among populations increased at the two highest temperatures (Figure 6). OF had the highest mortality among all population at 20°C (hazard ratio relative to LSI = 2.98 (95% CI: 1.20–7.44), *z* = 2.35, *p* = .02). Differences among populations increased further at 25°C, particularly in OF and ERI (Figure 6). The two southernmost populations (Ås and DK), in contrast, showed low mortality rates at all temperature treatments. A small increase in mortality was found at 25°C with increase in latitude (Figure 6, Table 4). Increasing values of *T*_S_ and dd caused small but significant reduction in the plasticity of mortality rates between 20 and 25°C (Table 4).

**Table 4:** Summary of the effects of population covariates on survival and differences among the five populations of *Folsomia quadrioculata*, estimated from Cox proportional hazards models. Effects of latitude, temperature of growing season (*T*_s_), and heat sum (dd) on variation among populations at 20°C and 25°C, and on interaction with temperature (20–25°C) (i.e., "Plasticity"), were estimated from separate models for each covariate. In the lower half, differences among populations (relative to LSI) are summarized for 25°C, where these differences were the largest. Due to little mortality at 10 and 15°C (<10%), these two temperatures have not been summarized below

Population covariates	Temperature		Plasticity^b^
	20°C^a^	25°C^a^	
Latitude (per 10°N)	0.83 (0.58, 1.18)	1.27 (1.02, 1.60)*	1.08 (0.99, 1.17)
*T*_S_ (per °C)	1.05 (0.99, 1.11)	0.97 (0.94, 1.00)	0.98 (0.97, 0.999)*
dd (per 100 day degree)	1.02 (0.99, 1.10)	0.98 (0.96, 1.00)	0.90 (0.98, 1.00)*

**Population differences at 25°C**			
Population	Hazard ratio (95% CI)	*z*	Hazard ratio of LSI
DK	0.18 (0.05, 0.60)**	−2.80	5.58
OF	1.65 (0.95, 2.89)	1.78	0.60
ERI	1.17 (0.59, 2.32)	0.46	0.85
Ås	0.07 (0.01, 0.54)*	−2.56	13.69

^a^Hazard ratio corresponding to unit increase in population covariate at 20 or 25°C.

^b^Interaction between a population covariate and temperature across 20 and 25°C.

*p < .5, **p < .01.

## 4 DISCUSSION

Our common garden experiments revealed substantial genotypic differences among populations of *F. quadrioculata* in most of the traits studied, reflecting considerable adaptability. However, the macroclimate variables could not be interpreted as the major climate‐related drivers of the responses of each trait. Instead, the traits revealed population‐specific differences, consistent with our previous study on embryonic traits of this species. (Sengupta et al., 2016). Life history tactics under specific conditions are shaped by coevolution of several traits (e.g., Stearns, 1976). Consequently, the present state or modification of one trait may affect responses of the other traits, emphasizing the importance of considering multiple life history traits for improving our understanding of the underlying selective forces (e.g., Forsman, 2014).

The population differences in reaction norms, including both trait means and phenotypic plasticity, were clearly trait‐specific. Age at first reproduction showed distinct population‐specific differences in trait means, but relatively small variation in plasticity. By comparison, the other life history traits varied substantially in plasticity among populations. For several traits, the population‐specific thermal reaction norms varied from linear to differentially curved lines. These findings are consistent with those of other studies emphasizing that the shapes of the response curves are prone to selection (Huey & Kingsolver, 1989; Klepsatel et al., 2013). The patterns of variation in plasticity among populations, indicated by differences in curvature of the reaction norms, emphasize the importance of evaluating thermal adaptation across several temperatures spanning a realistic temperature range.

When comparing the set of thermal reaction norms studied, it becomes evident that the response patterns were shaped by a complex set of climate‐related drivers. Only two traits showed significant effect of macroclimate variables. Firstly, age at first reproduction increased in phenotypic plasticity with increasing latitude, and decreasing summer temperature and annual heat sum. This agrees with the expectation that increasing development rates would enable populations from higher latitudes to utilize short spans of favorable conditions, such as warm spells, thereby compensating the time constraints imposed by short‐growing seasons (Conover & Schultz, 1995; Gotthard & Nylin, 1995). Secondly, the two southernmost populations (DK and Ås) from sites with relatively warm summer climate survived best at the two highest temperatures.

Undoubtedly, large climatic differences between the high arctic and the temperate sites represent important factors affecting performance of the populations studied. However, the low support for explanations relating genotypic variation to macroclimate calls for alternative explanations linked to local conditions. Microclimate of the ground can vary greatly even at local scales, depending on factors such as vegetation, topography, wind exposure, soil thickness, and humidity (e.g., Geiger, Aron, & Todhunter, 2003). In addition, differences in seasonality and climate stochasticity affect life history traits of animals (e.g., Dixie, White, & Hassall, 2015; Forrest & Miller‐Rushing, 2010; Sengupta et al., 2016; Tuljapurkar, Gaillard, & Coulson, 2009). In agreement with these arguments, most of the traits studied in *F. quadrioculata* showed population‐level differences that were consistent with climate predictability and seemingly contrasting microclimates of the selected sites (Appendix S1), as argued below.

1. The Danish population (DK), appeared to perform equally well across temperatures, with no apparent optimal temperature. Such a “jack of all trades, master of none” type of life history strategy (Huey & Hertz, 1984; Palaima & Spitze, 2004) appears suitable for this multivoltine population. It remains reproductively active throughout the year (Haarløv, 1960), thereby exposing all life history traits and stages of the life cycle to very different temperatures, depending on season (Rasmussen et al., 1982).
2. In contrast, the grass field population (Ås) is usually exposed to several months of frozen ground (Norwegian Meteorological Institute), and a 1‐year life cycle with distinct phenology has previously been described in another population from the same area (Leinaas, 1978). During the growth season, this open habitat would experience higher temperature and longer periods of dry conditions than forested areas would. This is consistent with the high survival of this population at the highest temperature treatment. Moreover, low reproduction, starting at an older age and larger size, together with previously documented large offspring size of this population (Sengupta et al., 2016) might be an adaptation to improve protection against desiccation (Kærsgaard, Holmstrup, Malte, & Bayley, 2004; Le Lagadec, Chown, & Scholtz, 1998).
3. In the Oslo Forest population (OF), all traits, except size at first reproduction, indicated specialization to a narrower and fairly low temperature range than that in the other populations, with an optimum around 15°C. In addition, this population showed the clearest signs of heat stress at the highest temperatures studied. This agrees with its predictable, seasonal environment, characterized by dense canopy and understory that prevent soil heating owing to insolation (Bjor, 1972).
4. The high arctic LSI showed among the fastest juvenile growth rates, early onset of reproduction, and high survival up to 20°C. These characteristics, together with high reproduction at 20°C, emphasized its ability to utilize fairly high temperatures, and perform reasonably well at low temperatures, in line with several arctic arthropods (Danks, 1999, 2004). Thus, similar to DK, LSI appears to be a temperature generalist, although for a different reason. It comes from a site characterized by highly unpredictable climate (Birkemoe & Leinaas, 2001; Sengupta et al., 2016). Consequently, the advantage of efficiently utilizing unpredictable warm spells may prevent typical cold adaptation in these traits (Clarke, 2003).
5. The other high arctic population (ERI) had large body size and high reproduction at 20°C, but little or no reproduction at lower temperatures, even though it inhabits by far the coldest site of this study (Sengupta et al., 2016). Apparently, the results were confounded by some additional factors not studied here. The predictably short and cool summer of Ellef Ringnes Island (McAlpine, 1965; Savile, 1961) is likely to have favored recruitment early in the growth season. In Collembolans, phenology is usually determined by a diapause that is terminated by winter cold (Leinaas & Bleken, 1983). Lack of an appropriate cold exposure to terminate the diapause could explain the reproductive failure at 10 and 15°C, whereas the unrealistically high temperature of 20°C may have decoupled the reproductive diapause without cold exposure. In addition, these distinct differences from the other populations might be related to the long‐term genetic isolation from the other Palearctic populations (Chahartaghi‐Abnieh, 2007; L. Deharveng, personal communication). However, the links between phylogeny and life history of this species are yet to be investigated in detail.

Correlations of macroclimate with life history characteristics have been shown in numerous highly mobile insect species (Barton et al., 2014; Blanckenhorn & Demont, 2004; Nygren, Bergstrom, & Nylin, 2008). By comparison, *F. quadrioculata* has very low dispersal ability (Hertzberg & Leinaas, 1998; Hertzberg et al., 1994), which increases the potential for adaptation to local conditions (Cassel‐Lundhagen, Kaňuch, Low, & Berggren, 2011; Lenormand, 2002; Slatkin, 1987). However, the role of climate in shaping thermal adaptation in life history traits of different soil‐dwelling life forms with low dispersal ability has remained relatively understudied. In addition, interpretation of the combined effects of macroclimate, and microclimate or habitat conditions on thermal adaptation might not be straightforward (e.g., Manenti et al., 2017) and requires extensive support from several other research approaches.

Our arguments about local adaptation are based on the general rejection of the macroclimate hypotheses, and the support for alternative explanation is based on existing information on other habitat and climate‐related factors of the sampling sites. However, to improve our understanding of the drivers underlying thermal adaptation in *F. quadrioculata* and other soil and litter‐dwelling species with limited dispersal ability, it would be of great interest to compare populations from contrasting habitat types within and across climatic zones, together with detailed records of microclimate parameters and demography from the different sites.

## CONFLICT OF INTEREST

None declared.

## AUTHOR CONTRIBUTIONS

SS and HPL conceived the idea, designed and performed the experiments, and wrote the manuscript. SS and TE analyzed the data. TE also helped in writing and improving the manuscript.

## DATA ACCESSIBILITY

The datasets used in this study have been archived in DRYAD with the following DOI: doi:10.5061/dryad.8dm60
