# Supplementary material for: Thermal plasticity in postembryonic life history traits of a widely distributed Collembola: Effects of macroclimate and microhabitat on genotypic differences
Source: Ecol Evol. 2017 Sep 5;7(19):8100–12. doi: 10.1002/ece3.3333 (PMC5632673; doi:10.1002/ece3.3333)
Supplement: Supplementary file 1 [file ECE3-7-8100-s001.docx]

**Appendix S1: Soil microclimates**

In the high arctic, sparse vegetation cover on the thin layer of biologically active soil provides little protection from insolation or cold winds, and prevailing weather conditions of an area may strongly affect the microclimate. Continuous sunshine during clear summer days can heat up the ground much above ambient air temperature (Coulson *et al.* 1996, Hodkinson *et al.* 1996, Danks 1999). The northernmost Little Slate Island (LSI) population inhabits the most stochastic thermal environment of the five populations, with a generally cold climate, and unpredictable periods when the ground is heated by sunny spells that differ greatly in duration between years (Sengupta, Ergon & Leinaas 2016). By contrast, the Ellef Ringnes Island population (ERI) has the most severe cold climate with low summer temperatures, typically characterised by fog and heavy cloud cover (Savile 1961; McAlpine 1965). Thus, with little insolation, conditions for soil-dwelling animals at this site are predictably cool throughout the growth season. The Oslo Forest population (OF) has the most predictable microclimate of the populations studied, with soil temperature fluctuations greatly damped by the dense canopy, and total coverage of understory and moss layer (Bjor 1972). It has a one-year life cycle and a distinct phenology with recruitment mainly in late spring and early summer (Leinaas 1978). Preliminary data suggest that the Ås population from a grass field close to Oslo also has a one-year life cycle. However, compared to the coniferous forest of OF, the snow free growth season is longer and diurnal temperature fluctuations are stronger (Schnug *et al.* 2014). Lastly, the Danish site usually has little snow and frost during winter (Rasmussen*,* Nielsen & Hansen 1982), allowing the DK population to be active most of the year (H. Petersen, pers. obs.). Thus, although living in a quite predictable seasonal environment, several generations per year (Haarløv 1960) lead to exposure of the sensitive juveniles to very different temperature conditions depending on season.

References

Bjor, K. (1972) Micro-temperature profiles in the vegetation and soil surface layers on uncovered and twig covered plots. *Meddelelser fra det Norske Skogforsøksvesen*, **30**, 199–218.

Coulson, S., Hodkinson, I., Wooley, C., Webb, N., Block, W., Worland, M., Bale, J. & Strathdee, A. (1996) Effects of experimental temperature elevation on high-arctic soil microarthropod populations. *Polar Biology*, **16**, 147–153.

Danks, H.V. (1999) Life cycles in polar arthropods — ﬂexible or programmed? *European Journal of Entomology*, **96**, 83–102.

Hodkinsonm, I.D., Coulson, S.J., Webb, N.R. & Block, W. (1996) Can high Arctic soil microarthropods survive elevated summer temperatures? *Functional Ecology*, **10**, 314–321.

Leinaas, H.P. (1978) Seasonal variation in sampling efficiency of Collembola and Protura. Oikos, **31**, 307–312.

McAlpine, J. (1965) Insects and related terrestrial invertebrates of Ellef Ringnes Island. *Arctic*, **18**, 73–103.

Rasmussen, S., Nielsen, M.K. & Hansen, J.P.N. (1982) The climate of a Danish beech wood, Hestehaven, eastern Jutland. *Holarctic Ecology*, **5**, 412–419.

Savile, D.B.O. (1961) The botany of the northwestern Queen Elizabeth Islands. *Canadian Journal of Botany*, **39**, 909–942.

Schnug, L., Jensen, J., Scott-Fordsmand, J.J. & Leinaas, H.P. (2014) Toxicity of three biocides to springtails and earthworms in a soil multi-species (SMS) test system. *Soil Biology and Biochemistry*, **74**, 115–126.

Sengupta, S., Ergon, T. & Leinaas, H.P. (2016) Genotypic differences in embryonic life history traits of *Folsomia quadrioculata* (Collembola: Isotomidae) across a wide geographical range. *Ecological Entomology*, **41**, 72–84.

**Appendix S2: Pre-experimental treatment**

All the experiments were performed from 2011–2013, *i.e.* 3–5 years (approx. 9–15 generations) after collection, ERI being the only exception (see below). The number of generations before the experiments is comparable to similar studies on much faster developing insects (*e.g.* Schmidt *et al.* 2005; Klepsatel *et al.* 2014).

Stocks of 500–1000 individuals per population were kept in 5–10 cultures with some mixing at least once a year (Sengupta 2015). The large number of founding individuals and large stock cultures with good reproduction and low mortality suggest little risk of the results being confounded by evolutionary changes during this period (Sengupta, Ergon & Leinaas 2016). Nonetheless, we tested the effects of culturing time on age at first reproduction in LSI and OF at 15°C in 2011 and 2013 (10 and 5 culture boxes respectively, for both populations). The measurements did not differ (log-linear models—LSI: t = 0.54, p = 0.60; OF: t = 0.78, p = 0.45), indicating no significant effect of culturing duration. Previously we have done similar tests on effect of culture period on hatchling size without finding any effect (Sengupta, Ergon & Leinaas 2016).

The ERI population was collected during an expedition in 1999, and it had not been feasible to re-sample it from this remote island. Consequently, by the time of the experiment, it had been reared in the lab considerably longer than the other populations. We chose to include this population from an extreme climate, as we believed it would add to our understanding of the evolution of life-history traits in the species. Early observations, from the first years after being sampled, indicated large differences from the other populations for thermal adaptations in egg development rate, reproduction patterns, and diapause strategy (Sengupta 2015; Sengupta, Ergon & Leinaas 2016). Owing to little reproduction in this population at 15°C (see Results), we tested for the effect of culturing time on age at first reproduction at 20°C, but found no significant effect (4 boxes each in 2011 and 2013; log-linear model: t = -0.25, p = 0.81).

References

Schmidt, P.S., Matzkin, L., Ippolito, M. & Eanes, W.F. (2005) Geographic variation in diapause incidence, life‐history traits, and climatic adaptation in *Drosophila melanogaster*. *Evolution*, **59**, 1721-1732.

Klepsatel, P., Gáliková, M., Huber, C.D. & Flatt T (2014) Similarities and differences in altitudinal versus latitudinal variation for morphological traits in *Drosophila melanogaster*. *Evolution*, **68**, 1385–1398.

Sengupta, S. (2015) *Life history traits and thermal adaptation in* Folsomia

quadrioculata *(Collembola) across climate regio*ns. PhD dissertation, Department of Biosciences, University of Oslo, Norway.

Sengupta, S., Ergon, T. & Leinaas, H.P. (2016) Genotypic differences in embryonic life history traits of *Folsomia quadrioculata* (Collembola: Isotomidae) across a wide geographical range. *Ecological Entomology*, **41**, 72-84.

**Table S1:** Summary of a log-linear mixed effects model comparing five populations of *F. quadrioculata* for body length at the age of 112 days at 10°C. Population was included as fixed effect and replicate boxes as a random effect. ‘Intercept’ in fixed effects refers to OF population.

Random effects:

SD

Replicates (Intercept) 0.0357

Residual 0.0991

Fixed effects:

Value SE DF t-value P

(Intercept) 0.3777 0.0247 176 15.2629 **0.0000**

Population DK -0.1358 0.0386 10 -3.5201 **0.0055**

Population LSI -0.1167 0.0367 10 -3.1771 **0.0099**

Population Ås -0.0905 0.0368 10 -2.4629 **0.0335**

Population ERI -0.1866 0.0357 10 -5.2223 **0.0004**

**Table S2:** Plasticity in juvenile growth rate (10–20°C) was analysed as follows. The length of an individual $i$ from population $p$ at age $A_{i}$ growing under temperature $T_{i}$ in replicate box $b\left( i \right)$ was modelled as a log-linear function:

$$\ln\left( L_{p}\left( A_{i},T_{i} \right) \right)=\alpha_{p}+\left( \beta_{p}^{\left( 0 \right)}+\beta_{p}^{\left( T \right)}T_{i} \right)A_{i}+\delta_{b(i)}+\varepsilon_{i}$$

where, $\alpha_{p}$ is the population specific log(hatching size) and $\beta_{p}^{\left( 0 \right)}+\beta_{p}^{\left( T \right)}T_{i}$ is the population-specific growth rate on a log scale as a function of temperature (reaction norm with intercept $\beta_{p}^{\left( 0 \right)}$ and slope $\beta_{p}^{\left( T \right)}$). Random variation among replicate boxes was modelled as a normal distribution with zero mean and standard deviation $\sigma_{b}$, $\delta_{b(i)}\sim N\left( 0,\sigma_{b}^{2} \right)$, and we assumed a normal distributed measurement error, $\varepsilon_{i}\sim N\left( 0,\sigma^{2} \right)$. $\beta_{p}^{\left( T \right)}$ summarised in the table below is thus a population specific measure of slope (plasticity). Populations not connected by the same letter do not have overlapping 95% CI.

Population $\beta_{p}^{\left( 0 \right)}$ 95% CI $\beta_{p}^{\left( T \right)}$ 95% CI

LSI -0.0011 -0.0043–0.0022^AB^ 0.0013 0.00108–0.00159^AB^

DK 0.0052 0.0014–0.0091^A^ 0.0008 0.00052–0.00109^A^

Ås -0.0022 -0.0056–0.0012^B^ 0.0012 0.00102–0.00154^AB^

OF -0.0008 -0.0037–0.0020^AB^ 0.0014 0.00120–0.00166^B^

ERI -0.0068 -0.0097– -0.0040^AB^ 0.0016 0.00143–0.00190^B^

$\beta_{p}^{\left( T \right)}$ did not show significant correlation with latitude (Pearson’s r = 0.61 (95% CI: -0.57 –0.97), t = 1.41, df = 3, p = 0.25), T_S_ (Pearson’s r = -0.56 (95% CI: -0.97–0.63), t = -1.18, df = 3, p = 0.32) or dd (Pearson’s r = -0.60 (95% CI: -0.97–0.60), t = -1.28, df = 3, p = 0.29)

**Table S3:** Population-specific asymptotic size at four temperatures estimated by the von Bertalanffy growth model fitted to mean length of animals in each replicate box. Due to slow growth and linearity of the growth curves, asymptotic size could not be estimated for two populations at 10°C.

| Population | Temperature | Mean | 95% CI |
| --- | --- | --- | --- |
| LSI | 10 | 2.11 | 1.61–2.61 |
| LSI | 15 | 1.68 | 1.60–1.75 |
| LSI | 20 | 1.44 | 1.37–1.50 |
| LSI | 25 | 1.21 | 1.09–1.34 |
| ERI | 10 | NA | NA |
| ERI | 15 | 1.88 | 1.53–2.23 |
| ERI | 20 | 1.99 | 1.63–2.36 |
| ERI | 25 | 1.13 | 0.97–1.30 |
| OF | 10 | 3.25 | 1.67–4.83 |
| OF | 15 | 1.96 | 1.80–2.11 |
| OF | 20 | 1.43 | 1.35–1.50 |
| OF | 25 | 1.02 | 0.91- 1.13 |
| Ås | 10 | NA | NA |
| Ås | 15 | 2.50 | 1.24–3.76 |
| Ås | 20 | 1.52 | 1.29–1.75 |
| Ås | 25 | 1.43 | 1.07–1.79 |
| DK | 10 | 2.81 | 0.78–4.83 |
| DK | 15 | 1.59 | 1.29–1.89 |
| DK | 20 | 1.54 | 1.30–1.79 |
| DK | 25 | 1.29 | 1.12–1.45 |

**Table S4:** Correlation (Pearson’s coefficient) of estimated population specific asymptotic size and size at first reproduction at each treatment temperature with latitude, T_S_, and dd. 95% CI are provided within brackets. Note that at 10°C asymptotic size could be estimated for three out of the five populations (Fig. 2) and size at first reproduction could be estimated for four populations (Fig. 4b). None of the correlations was significant.

| Asymptotic size | | | |
| --- | --- | --- | --- |
|  | 10°C | 15°C | 20°C |
| Latitude | -0.54 (-) | -0.33 (-0.93, 0.77) | 0.39 (-0.75, 0.95) |
| T_S_ | 0.69 (-) | 0.42 (-0.73, 0.95) | -0.51 (-0.96, 0.67) |
| dd | 0.69 (-) | 0.36 (-0.77, 0.94) | -0.47 (-0.96, 0.70) |
| (p > 0.37) | | | |
| Size at first reproduction | | | |
| Latitude | -0.28 (-0.98, 0.93) | 0.38 (-0.76, 0.94) | 0.12 (-0.85, 0.91) |
| T_S_ | 0.45 (-0.90, 0.98) | -0.43 (-0.95, 0.73) | -0.01 (-0.88, 0.88) |
| dd | 0.37 (-0.92, 0.98) | -0.42 (-0.95, 0.73) | -0.05 (-0.89, 0.87) |
| (p > 0.47) | | | |


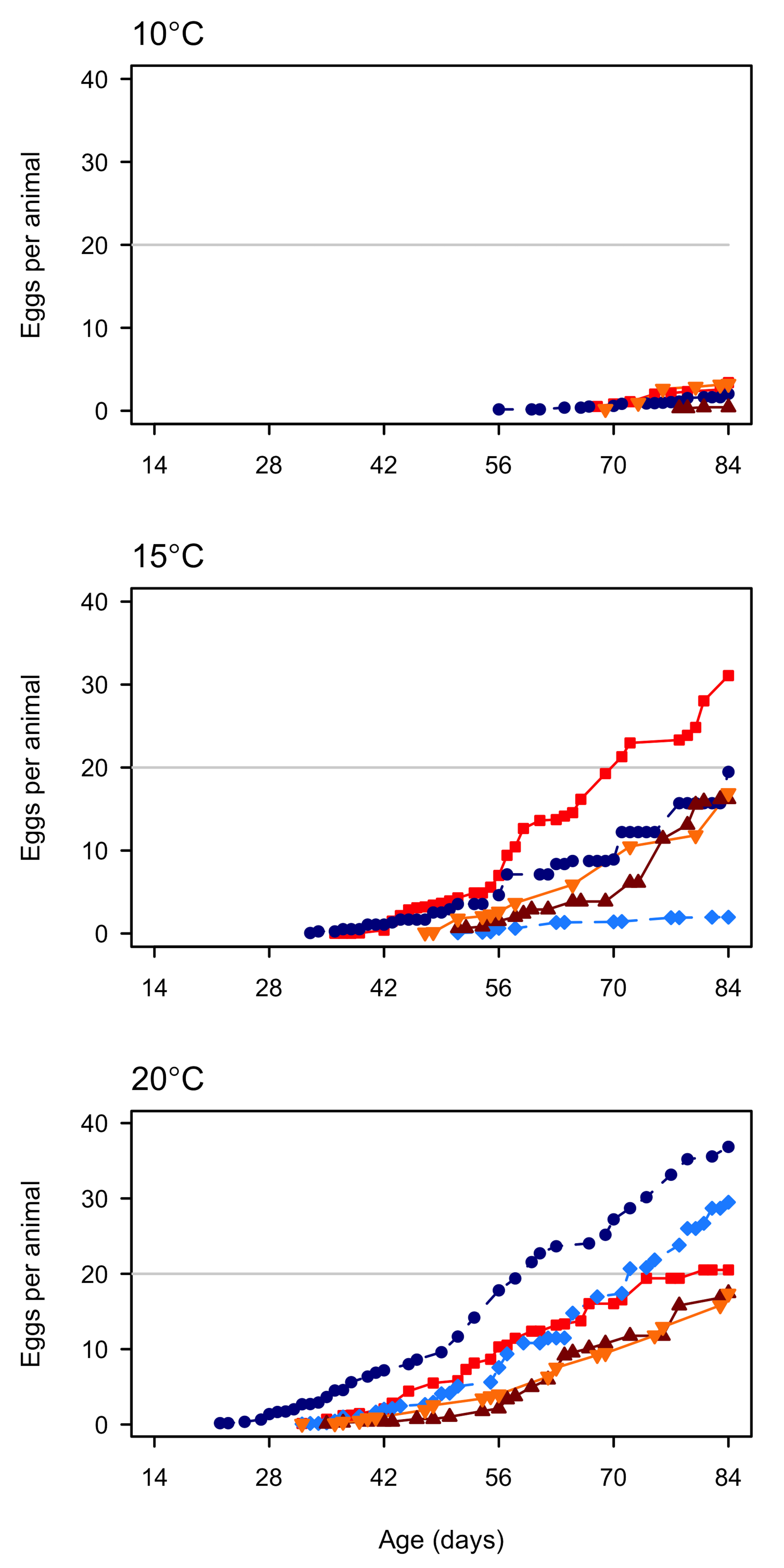


**Fig. S1:** Fecundity (cumulative sum of eggs per animal alive) plotted against age (in days). Populations are indicated by different colours and shapes: dark blue circles–LSI, light blue diamonds–ERI, bright red squares–OF, dark red triangles–Ås, and yellow inverted triangles–DK. The ERI population did not reproduce at 10°C and none of the populations reproduced at 25°C. The grey line corresponds to 20 eggs per animal.
